# New Hospital for Women

**Published:** 1916-12-23

**Authors:** 


### WOMEN IN MEDICINE. 1866—1916.

### New Hospital for Women.

To commemorate the arrival of the New Hospital for Women at its fiftieth year of beneficent activity, a crowded and enthusiastic meeting was held on Tuesday afternoon, the 12th inst., in a large marquee adjoining the hospital building in Euston Road. We published Sir Alfred Keogh's address in our issue of December 16 (see p. 217).

**Mrs. Mary Scharlieb, M.D., M.S.,** said that in St. Paul's Cathedral stood a monument to Sir Christopher Wren, on which was an inscription bidding those who sought a monument to the famous architect to look around. This hospital, which was to-day celebrating its jubilee, was not, happily, a monument, but was a memorial to its founder, Mrs. Garrett Anderson, who was a good and true woman. She, the speaker, was associated with that lady at this hospital thirty years ago, and perhaps she knew, better than did the juniors, with what wisdom and self-abnegation Mrs. Anderson laid the foundation not only of this material hospital, but of what was really its spirit and soul. If one needed a monument to her, one had only to look around at all the medical women who were now doing good work throughout the world. There may have been surgeons as skilful, or more skilful; there may have been pathologists more scientific and more prolific in good work, but they had never had among them any other woman with such a statesmanlike outlook and such power of using all with whom she came into contact. This hospital had done wonders; it had supplied the great missionary societies, to a large extent, with their medical women; and it was at the present time supplying not only the Military Hospital in Endell Street, but also military hospitals in France, Serbia, in Southern Russia, and other parts of the war area. Their medical women were also to be found in many of the large Government Departments; they were also serving on the female side of lunatic asylums and of prisons. Only a few days ago she heard Mr. Ernest Lane say he had never known the work of a certain hospital so well done as when one of their junior women was a house-surgeon on the staff.

**Sir John Bland-Sutton** said many great movements which had been introduced for the benefit of mankind had been ill-judged, because those who ill-judged them were rarely honest enough to look fairly and squarely into the facts. A few years after this hospital was built he used to visit it occasionally, and those visits were of great advantage, because he formed the acquaintance of some members of the staff, and notably Mrs. Garrett Anderson and Mrs. Scharlieb. Their confidence, their ability, and, above all things, their zeal, made a very profound impression on his mind. Many people thought one had only got to found a hospital and open the doors, and that it would be immediately filled with patients. No such thing. Thirty-four years of hospital life had taught him that the hospital patient was a curiously discriminating person. If the hospital were good, the out-patient department would be crowded, the wards would be filled, and the waiting-list would be a long one. This hospital did good work; he knew it. Some years ago, he was interested in a particular set of operations, and he used to obtain the annual reports from the secretary. He had read and admired those reports. And he had obtained reports from those hospitals in London which furnished reports—for all did not publish reports—and from all those he had drawn up comparative statistics of the particular operations and published them. He was pleased to say that the results of the gynæcological operations at this hospital were as good as any in London, and beaten by none. He had listened to papers by women at medical societies which admit

(*Continued on p. iv.*)

